# Japanese Encephalitis Outbreak, Yuncheng, China, 2006

**DOI:** 10.3201/eid1307.070010

**Published:** 2007-07

**Authors:** Li-Hua Wang, Shi-Hong Fu, Huan-Yu Wang, Xiao-Feng Liang, Jing-Xia Cheng, Hong-Mei Jing, Gen-Lao Cai, Xing-Wang Li, Wen-Yuan Ze, Xin-Jun Lv, Hua-Qing Wang, Ding-Lin Zhang, Yun Feng, Zun-Dong Yin, Xiao-Hong Sun, Tie-Jun Shui, Ming-Hua Li, Yi-Xing Li, Guo-Dong Liang

**Affiliations:** * Institute for Viral Disease Control and Prevention, Beijing, People’s Republic of China; † Chinese Center for Disease Control and Prevention, Beijing, People’s Republic of China; ‡ Shanxi Center for Disease Control and Prevention, Taiyuan, People’s Republic of China; § Yuncheng Center for Disease Control and Prevention, Yuncheng, People’s Republic of China; ¶ Yuncheng Infectious Diseases Hospital, Yuncheng, People’s Republic of China. #Beijing Ditan Hospital, Beijing, People’s Republic of China; ** Beijing Institute of Biological Products, Beijing, People’s Republic of China

**Keywords:** Viral encephalitis, Japanese encephalitis virus, outbreak, genotypes, China, letter

**To the Editor:** Japanese encephalitis (JE) epidemics have occurred only in Asia. More than 50,000 cases of JE with ≈10,000 deaths have been reported since 1998 (*1**,**2*). The People’s Republic of China reported 5,104 cases and 214 deaths in 2005. Most of these deaths occurred in infants (*3**,**4*).

During July and August 2006, an outbreak of viral encephalitis occurred in Yuncheng, Shanxi Province, People’s Republic of China. A total of 66 cases (1.32/100,000 population) were reported, including 19 deaths (case-fatality rate 28.8%). The cases had a widespread distribution over 9 counties and involved 37 towns and 61 administrative villages. The ratio of male-to-female patients was 1:0.89. A distinct clinical feature of this outbreak was the age distribution. More than 86% of the patients were >30 years of age, with only 10% of patients <7 years of age; ≈95% of the deaths occurred in patients >50 years of age (*5*).

We report serologic and virologic findings for the 2006 outbreak of viral encephalitis. Forty-six clinical specimens collected from 34 patients who had a diagnosis of viral encephalitis, including 33 serum samples and 13 cerebrospinal fluid (CSF) samples, were studied. All serum samples were screened for immunoglobulin M (IgM) to West Nile virus (WNV) by using the WNV IgM-capture ELISA kit (PanBio, Brisbane, Queensland, Australia) and for IgM to dengue virus or Japanese encephalitis virus (JEV) by using the JE-Dengue IgM Combination ELISA kit (PanBio). Results for JEV were confirmed by using the JE Virus IgM-Capture ELISA kit (Shanghai B & C Enterprise Development Co. Ltd, Shanghai, People’s Republic of China).

WNV-specific or dengue virus–specific IgM was not detected in any samples. JEV-specific IgM was detected in 27 (80%) patients, which indicated recent JEV infections. The other 7 patients were negative for JEV by ELISA and reverse transcription–PCR (RT-PCR). Increases >4-fold in neutralizing antibodies were detected in acute- and convalescent-phase serum samples from 9 patients (10 serum pairs were collected during the outbreak).

Attempts were made to detect virus in CSF of patients and in 2,400 mosquitoes. Mosquitoes (mainly *Culex* spp.) were collected in cow sheds and hog pens around houses and processed into pools of 100. Total RNA was extracted from CSF or mosquito homogenate by using the QIAamp viral RNA extraction kit (QIAGEN, Valencia, CA, USA) according to the manufacturer’s specifications. RT was performed by using Ready-To-Go-You Prime First Strand Beads (Amersham Pharmacia Biotech, Piscatawy, NJ, USA) and a seminested PCR to amplify 492-bp gene fragments of the premembrane (PrM) sequence of JEV by using the Takara LA Taq PCR kit (Takara Bio Inc., Shiga, Japan). The primers were derived from Ishikawa strain genome sequences (GenBank accession no. AB051292). Primers PrMF: 5′-CGT TCT TCA AGT TTA CAG CAT TAG C-3′ (251–275), PrMR1: 5′-CGY TTG GAA TGY CTR GTC CG-3′ (724–743), and PrMR2: 5′-CCY RTG TTY CTG CCA AGC ATC CAM CC-3′ (901–925) were used.

JEV PrM gene was amplified from CSF of 6 (46%) of 13 patients and 10 of 24 pools of mosquitoes by using the same seminested RT-PCR. To identify JEV genotype(s) involved in this outbreak, PCR products were sequenced. Eleven sequences (GenBank accession nos. EF434264–EF434274) were obtained from 6 patients and 5 pools of mosquitoes. The 11 sequences were compared phylogenetically with17 known JEV strains of the 4 recognized genotypes (classified on the basis of a 240-nt region of the prM gene). As shown in the Figure, the 11 sequences were those of JEV.

**Figure:**
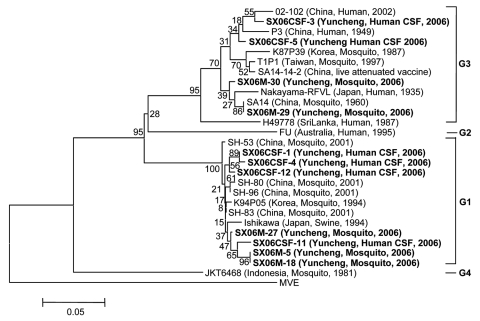
Phylogenetic analysis of Japanese encephalitis virus strains predicted from premembrane gene sequences. Neighbor-joining tree was generated by using MEGA 3.1 software (www.megasoftware.net) and rooted with Murray Valley encephalitis (MVE) virus sequence information. Bootstrap confidence limits for 1,000 replicates are indicated above each branch. Horizontal branch lengths are proportional to genetic distance; vertical branch lengths have no significance. Scale bar indicates no. nucleotide substitutions per site. All sequences from this study are in **boldface**. Genotypes are indicated on the right. Designations are listed first, followed by country, source, and year of isolation. CSF, cerebrospinal fluid.

Further analysis showed that these 11 sequences can be grouped into genotypes I and III. Both genotypes were found in patient and mosquito samples, indicating that these genotypes co-circulated during this JE outbreak.

JE has been endemic in Yuncheng for many years (*6*). A vaccine against JE (SA14–14–2) has been used in this area in infants, but not in adults. This might be 1 reason why a higher adult incidence was found in this outbreak. JEV genotype III had been the predominant genotype in previous years, but genotype I has been recently detected at increased frequencies (*7**–**10*). Detection of 2 JEV genotypes in 1 epidemic has not been reported. Whether simultaneous circulation of >1 genotype during an outbreak indicates a new type of emergence of JEV or that this has occurred and not been detected is unknown.
